# Regenerative endodontics: a true paradigm shift or a bandwagon about to be derailed?

**DOI:** 10.1007/s40368-016-0265-5

**Published:** 2017-01-13

**Authors:** H. Nazzal, M. S. Duggal

**Affiliations:** Department of Paediatric Dentistry, Leeds School of Dentistry, University of Leeds, Clarendon Way, Leeds, LS2 9LU UK

**Keywords:** Non-vital immature teeth, Regenerative endodontics, Revascularisation

## Abstract

**Aims:**

Regenerative endodontic techniques (RETs) have been hailed as a paradigm shift for the management of traumatised non-vital immature permanent anterior teeth. In this article the aim was to critically appraise the literature with regards to the outcome of regenerative endodontics on root development.

**Methods:**

Critical review of the literature where regenerative endodontic techniques have been used in the management of immature non-vital teeth with continuation of root development as the main outcome reported.

**Results:**

Most studies published were in the form of case reports and series with very few randomised controlled trials with a high risk of bias. Continuation of root development following the use of RET has been shown to be unpredictable at best with lower success in those teeth losing vitality as a result of dental trauma.

**Conclusions:**

Despite the high success of regenerative endodontics in terms of periodontal healing including resolution of clinical and radiographic signs and symptoms of infection, continuation of root development remains an unpredictable outcome. The use of a blood clot as a scaffold in regenerative endodontics should be reviewed carefully as that might offer an environment for repair rather than regeneration. In addition, preservation of structures, such as Hertwig’s epithelial root sheath, may have an important bearing on the success of this approach and should be further investigated.

## Introduction

**Fig. 1:**
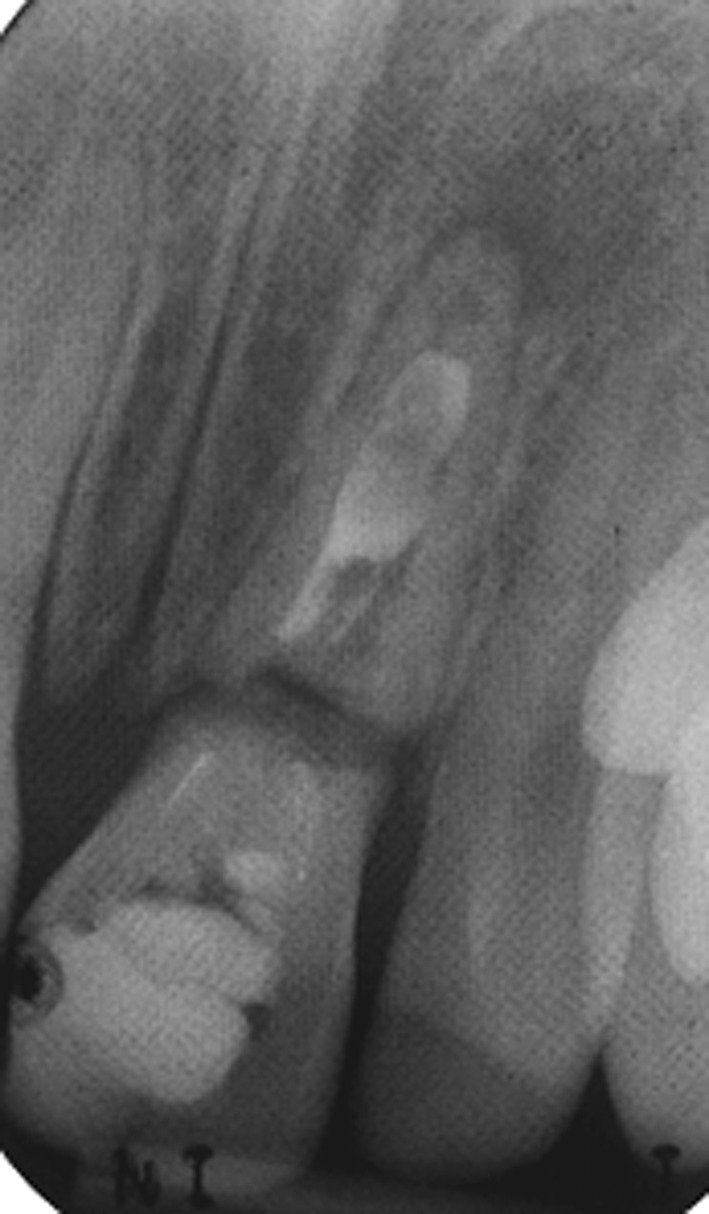
Periapical radiograph showing cervical root fracture of a tooth treated with calcium hydroxide apexification

Management of non-vital immature anterior permanent teeth in children remains a challenge in paediatric dentistry and endodontics. Once the tooth becomes non-vital, root development ceases, rendering the tooth weak and unable to withstand physiological forces of mastication. This results in a high root fracture rate with poor prognosis in the medium to long term. Indeed most studies have shown that over 50% of such teeth will be lost in the first 10 years following trauma despite being endodontically treated (Cvek 1992; Andreason et al. 2002; Al-Jundi 2004). Traditional endodontic treatment approaches have concentrated on achieving disinfection followed by creation of an apical barrier against which the root filling can be condensed. This has been achieved using either an apexification approach with the use of calcium hydroxide, or more recently with the use of mineral trioxide aggregate (MTA) to physically create a barrier against which the root canal can be obturated with a root filling material, such as gutta percha. Both these techniques have fundamental problems in that although they allow root canal obturation, they do not contribute to any quantitative or qualitative increase in root dimensions, and ultimately suffering root fractures (Cvek 1992; Andreason et al. 2002) leaving the child with a treatment burden for the rest of their lives (Fig. 1).

In order to achieve any quantitative or qualitative increase in root dimensions, it would be essential to restore the blood supply to the tooth that was disrupted in the first place. More recently there has been a paradigm shift in the management approach of this intractable clinical problem. Regenerative endodontic techniques (RETs) have been recently used with the ultimate aim of inducing further root development and thickening of root dentinal walls. There is now a better understanding of tissue engineering of the pulp-dentine complex that may make it possible to design appropriate RETs and technologies in order to achieve regeneration of the pulp-dentine complex (Murray et al. 2007).

**Fig. 2:**
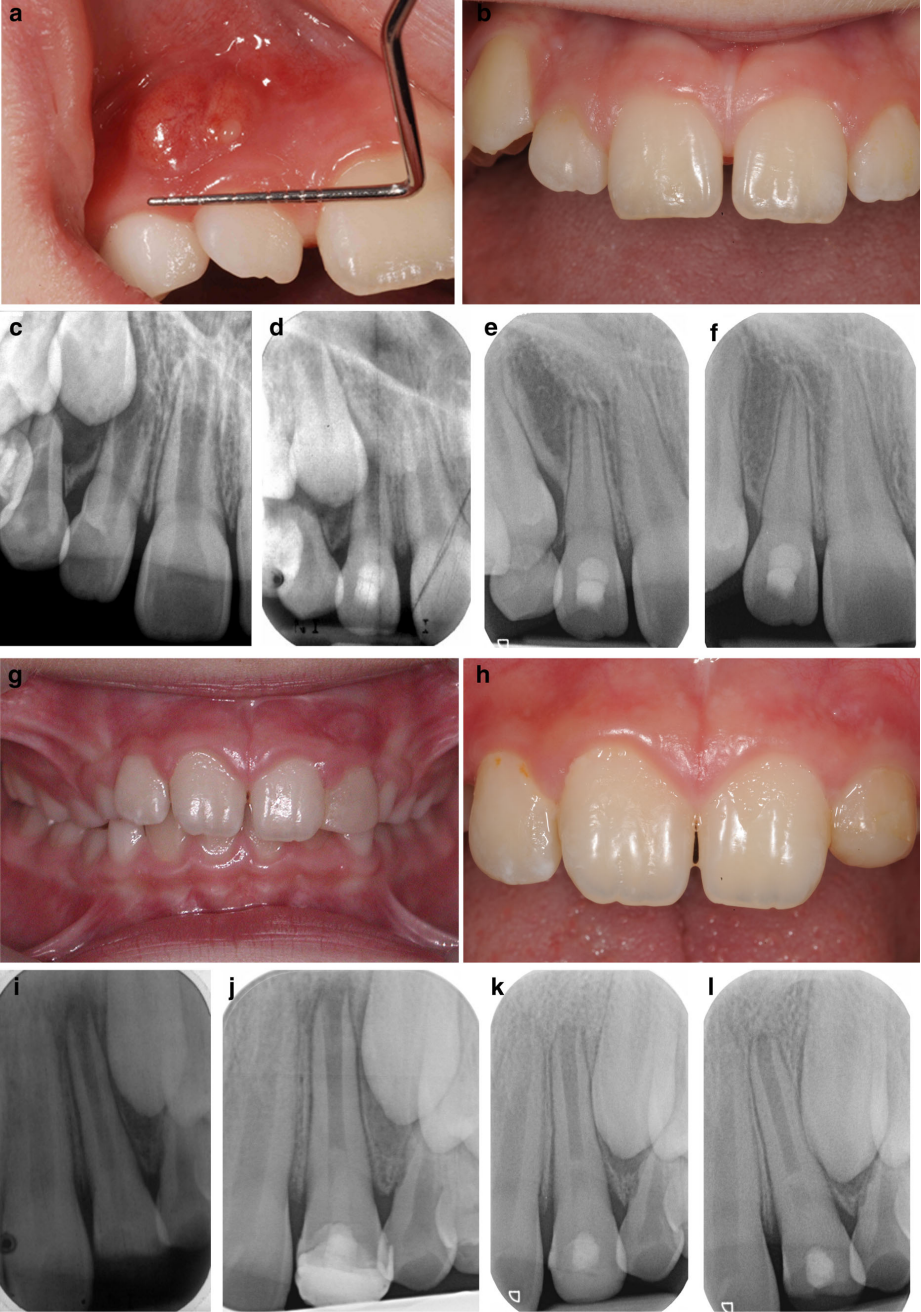
Clinical photographs and radiographic examination of two cases treated with regenerative endodontic technique showing success and survival outcomes. **a** Photograph showing labial abscess with discharging sinus related to the non-vital 12 secondary to dens invaginatus. **b** Photograph showing resolution of signs of infection (swelling and discharging sinus) maintained for up to 24 months following RET treatment of 12. **c**–**f** Periapical radiographs taken at baseline (showing an immature 12 with <1/2 root formation, thin dentinal walls and wide open apex), and follow-up at 3 months, 9 months and 2 years showing complete success following RET with gradual root formation and thickening of dentinal root walls. **g** Photograph showing traumatised non-vital 21 which sustained an enamel/dentine fracture. **h** Photograph showing no signs of infection (swelling and/or discharging sinus) at 24 months following RET treatment of 21. **i**–**l** Periapical radiographs taken at baseline (showing an immature 21 with <2/3 root formation, thin dentinal walls and wide open apex), and follow-up at 3 months, 9 months and 2 years showing no evidence of periapical lesion and with no signs of continuation of root development nor thickening of dentinal walls. The apical root canal space (**l**) shows evidence of radiopaque trabeculation suggestive of bony ingrowth

A form of RET was described in the literature by Östby as early as (1961). However, the current technique only gained popularity as a treatment option for immature non-vital teeth in the last decade. It was thought that, if successful, the use of RET could, in addition to promoting healing of the periapical area and resolution of clinical symptoms and signs of infection, promote continuation of root development and thickening of dentinal walls. However, whenever a new technique is introduced into clinical practice, especially one that claims to represent a paradigm shift, it is imperative to critically assess the outcome and to study whether it had indeed lived up to the expectations. When assessing the results of treatments performed with this technique it is essential to carefully consider what constitutes success and survival. Success of RET should, in the best case scenario, deliver periodontal healing, continuation of root development and thickening of dentinal walls (Fig. 2a–f). Survival of the tooth, on the other hand, could include those cases where periodontal healing had occurred with no further continuation of root development and/or thickening of dentinal walls (Fig. 2g–i).

Several prospective published studies have described various techniques and reported conflicting results on the success and survival, with the most striking finding of only limited success with the use of this technique reported by most studies (Kontakiotis et al. 2014). Although periodontal healing is reported to be seen in 79–100% of cases, continued root development and dentinal wall thickening were achieved in far fewer cases (Table 1). The use of a mixture of antibiotics, which would be the effective against bacteria most commonly associated with root canal infections, meant that healing was an understandable and a predictable outcome. Therefore, despite the growing use of RET over the last decade, these techniques have thus resulted in tooth survival in the short term with questionable and inconsistent success in the medium to long terms (Table 1). Survival and success of RET is illustrated in the two cases presented in Fig. 2. In this figure a clear distinction of what might be considered as a successful RET case is presented where healing was accompanied by continuation of root development and thickening of dentinal walls in an immature UR2, which lost vitality due to a developmental defect (Fig. 2a–f). Survival, on the other hand (Fig. 2g–l) is considered where there are clinical and radiographic signs of complete healing but with no evidence of continuation of root development or thickening of dentinal walls. The apical root canal space showed evidence of radiopaque trabeculation suggestive of bony ingrowth.

**Table 1:** Studies published until Feb 2015 showing success of regenerative endodontic technique in terms of periapical healing, continuation of root development, thickening of dentinal walls and apical closure

Study	Aetiology	Age (years)	Groups T = Treatment C = Control	Follow-up (months)	Periapical healing	Continued root development	Dentinal walls thickening	Apical closure
Jung et al. (2008)	Caries = 1 Anomaly = 5 Others = 2	9–14	T = RET TAP BC (*n* = 4) *C* = RET Ca(OH)_2_ Collatape (*n* = 5)	12–24	100% (8/8)	62.5% (5/8)^a^	78% (6/8)^a^	62.5% (5/8)^a^
Shah et al. (2008)	Trauma = 14	9–18 (mean 11.6)	T = RET FC BC	6–42	93% (13/14) ++, +++ 78.6% (11/14) +	71% (10/14)	57% (8/14)	–
Bose et al. (2009)	Variable = 88	–	T1 = RET TAP UK T2 = RET Ca(OH)_2_ UK T3 = RET FC UK C1 = MTA C2 = NSRCT	0–>36	–	RET TAP and RET Ca(OH)_2_ produced significantly greater increases than MTA or NSRCT	RET TAP produced significantly greater differences than RET Ca(OH)_2_ or RET FC	–
Chueh et al. (2009)	Trauma = 1 Caries = 1 Dens evaginatus = 21	6.8–14.2 (mean: 11 ± 1.7)	T = RET Ca(OH)_2_ no scaffold	Short term = 6–30 Long term = 7–108	100%;	91.3% (21/23)	–	91.3% (21/23)
Ding et al. (2009)	Trauma = 5 Anomaly = 7	8–11	T = RET TAP BC	Minimum 12	100%^b^	100% (3/3)^b^	–	100% (3/3)^b^
Petrino et al. (2010)	Trauma = 4 Caries = 2	6–13	T = RET TAP BC + CollaPlug	9–12	100% (6/6)	50% (3/6)	83.3% (5/6)	16.7% (1/6)
Cehreli et al. (2011)	Caries = 6	8–11	T = RET Ca(OH)_2_ BC	9–10	100%	100% (6/6) 0% (0/6)^c^	100% (6/6) 83% (5/6)^c^	100%
Chen et al. (2012)	Caries = 3 Anomaly = 7 Trauma = 10	8–13	T = RET Ca(OH)_2_ BC	6–26	100%	75% (15/20)	100% Marked: 20% (4/20)	Complete = 75% (15/20) Blunt = 25% (5/20)
Dabbagh et al. (2012)	Trauma = 14 Caries = 2 Anomaly = 2	7–16	T = RET TAP BC	24	100% (9/9)	100% (16/16)	–	–
Jadhav et al. (2012)	Trauma = 20	15–28	T = RET TAP PRP + BC (n = 10) C = RET TAP BC (*n* = 10)	12	T = 70% ++ C = 40% ++ 50% +++	T = 10% + 50% ++ 40% +++ C=40% + 60% ++	T = 20% + 50% ++ 30% +++ C = 30% + 70% ++	T = 30% ++ 20% +++ C = 30% ++ 70% +++
Jeeruphan et al. (2012)	Caries = 9 Anomaly = 16 Trauma = 36	T:12.9 ± 5 C1: 14.6 ± 6 C2: 10.5 ± 3.8	T = RET TAP BC + Collaplug (*n* = 20) C1 = MTA(n = 19) C2 = Ca(OH)_2_ (*n* = 22)	T = 21 ± 12 C1 = 14 ± 8 C2 = 27 ± 30	T = 80% (16/20) C1 = 68.42% (13/19) C2 = 77.3% (17/22)	T = 14.9% C1 = 6.1% C2 = 0.4%	T = 28.2% C1 = 0% C2 = 1.52%	–
Jadhav et al. (2013)	Trauma = 6	10–23	T = RET TAP BC + PRP C = RET TAP BC	12	T = 2/3 +++ C = 3/3 ++	Comparable in both groups	T = 100% (3/3) ++ C = 100% (3/3) +	T = 33% (1/3) +++ C = 100% (3/3) +
McTigue et al. (2013)	Anomaly = 4 Trauma = 28	6–17	T = RET TAP BC	29 cases: 12–48 3 cases: <12	96.8%	65.6% (21/32)	68.8% (22/32)	71.9% (23/32)
Alobaid et al. (2014)	Trauma = 24 Caries = 4 Anomaly = 1	6–16	T = RET $ BC (*n* = 19) C = MTA (*n* = 12)	T = 14 ± 8.5 C = 21.8 ± 12	–	T = 0%^c^ C = 12.5% (1/8)^c^	T = 20% (3/15)^c^ C = 0%^c^	Not reported
Kahler et al. (2014)	Anomaly = 3 Trauma = 13	7–12	T = RET TAP BC	18	90.3%	33.3% (3/9) 11.1% (1/9)^c^	88.9% (8/9) 55.6% (5/9)^c^	Complete: 19.4% Incomplete: 47.2%
Nagata et al. (2014)	Trauma = 23	7–17	T1 = RET TAP BC (*n* = 12) T2 = CaOH_2_ (*n* = 11)	1–19	T1 = 100% T2 = 80%	T1 = 41.7% T2 = 27.3%	T1 = 41.7% T2 = 45.4%	T1 = 66.7% T2 = 54.5%
Nagy et al. (2014)	Trauma = 36	9–13	T1 = RET TAPD BC (*n* = 12) T2 = RET TAPD FGF (*n* = 12) C = TAPD MTA (*n* = 12)	3–18	T1 = 100% T2 = 90% C = 80%	–	–	T1 = 100% T2 = 90% C = 80%
Saoud et al. (2014)	Trauma = 20	Mean: 11.3 ± 1.9	T = RET TAP BC	12	90% (18/20)	5% (1/20) 0%^c^	21% (4/20) 45% (9/20)^c^	55% (11/20)
Bezgin et al. (2015)	Trauma = 14 Caries = 6	7–13	T = RET TAPC PRP (*n* = 10) C = RET TAPC BC (*n* = 10)	18	T = 100% (7/7) C = 88.9% (8/9)	–	–	T = 70% (7/10) C = 60% (6/10)
Narang et al. (2015)	Not reported	<20	C = MTA (*n* = 5) T1 = RET TAP BC (*n* = 5) T2 = RET TAP PRF (*n* = 5) T3 = RET TAP PRP collagen (*n* = 5)	18	C = 40% +++ 60% ++ T1 = 40%+ 60% ++ T2 = 98% +++ 2% ++ T4 = 20%+ 80% ++	C = 0% T1 = 60% + 40% ++ T2 = 100% +++ T3 = 60% + 40% ++	C = 0% T1 = 50% + 50% ++ T2 = 40% ++ 60% +++ T3 = 80%+ 20% ++	C = 0% T1: 33.3% ++ 66.6% +++ T2: 60% + 40% ++ T3: 40%+ 60% ++

+ Satisfactory, ++ good, +++ excellent, $ varying intra-canal medicament, *T* test group, *C* control group, *RET* regenerative endodontic technique, *BC* blood clot, *PRP* platelet rich plasma, *PRF* platelet rich fibrin, *TAB* tri-antibiotic paste (Ciprofloxacin, Minocycline, Metronidazole), *TAPC* tri-antibiotic paste (Ciprofloxacin, Minocycline, Cephaclor), *TABD* tri-antibiotic paste (ciprofloxacin, doxycycline, metronidazole), *Ca(OH)*_*2*_ calcium hydroxide, *FC* Ferric Sulphate, *MTA* mineral trioxide aggregate, *NSRCT* conventional RCT with gutta purcha, *GP* gutta purcha only, *FGF* blood clot and an injectable hydrogel scaffold impregnated with basic fibroblast growth factor, *UK* unknown scaffold

^a^Results were not very clear in the paper

^b^Very high dropout rate

^c^Results when a 20% or more increase in root dimension is deemed clinically significant

The present review aims to critically appraise the current literature with regards to the possible reasons for the lack of consistent results with some teeth showing excellent success while others are merely surviving.

One of the reasons for this inconsistency could be that the currently used techniques do not follow the fundamental principles of tissue engineering required for regeneration of a pulp-dentine complex. The aim, therefore, was to review the evidence around the currently used RETs in view of the known tissue engineering principles.

Another possible factor for the inconsistent outcomes for further root development following RET could be the degree of damage to Hertwig’s epithelial root sheath (HERS) secondary to dental trauma or during damage of the periapical area during the stage of induction of bleeding into the root canal. Earlier studies (Table 1) presented their results by combining all non-vital immature teeth regardless of the aetiology for the loss of vitality. For example an immature permanent incisor could become non-vital due to trauma or a development anomaly such as dens invaginatus. The pathogenesis of these two conditions is quite different, with differing levels of damage to HERS. The authors firmly believe that variable and inconsistent outcomes for continuation of root development and thickening of dentinal walls are being reported because clinicians have failed to make a distinction between the various reasons for loss of vitality, and therefore the degree of damage to the epithelial root sheath. The authors believe that this is a crucial factor that would determine the outcome for RET on whether a tooth would exhibit a healing outcome only (survival) or demonstrate a qualitative increase in root dimensions through an increase in dentinal wall thickness and further root development (success).

Finally a lack of standardised radiographic exposure and measurement methods could contribute to these conflicting results and will be briefly discussed.

## Tissue engineering principles and regenerative endodontic technique (RET)

A few different techniques have been proposed in the literature (Murray et al. 2007), with the primary aim of regenerating a new pulp-dentine complex. For any technique to be successful it should, in principle, fulfil four key elements of tissue engineering needed for successful regeneration: availability of stem cells, suitable scaffolds, favourable signalling molecules and achieving a sterile environment for the stem cells to regenerate. However all the published techniques have applied the principles of tissue engineering loosely. For example no stem cells or scaffold are actively placed in the root canals as part of this method, instead these techniques rely on inducing a blood clot within the root canal to serve as a scaffold and as a source of stem cells. There is little evidence that this method would indeed result in a biologically effective scaffold that would have the stem cells, required for regeneration, or indeed signalling molecules, required for targeted differentiation of the tissues. It is important to individually consider all the key elements required for regenerating a dentine-pulp complex and to critically evaluate the current regenerative techniques to assess whether they are realistically capable of creating these key elements required for its successful clinical application in patients.

### Sterilisation

Sterilisation of the canal is the only factor reliably under the control of clinicians. Sodium hypochlorite, with concentrations of 1–6%, have been used as the only irrigant or in combination with other irrigants in most of the studies reported on RET published till May 2014 (Kontakiotis et al. 2015). This irrigant has been shown to be a potent antimicrobial material that dissolves organic matter (Martin et al. 2014).

Some laboratory studies have investigated the effect of sodium hypochlorite on stem cells. Martin et al. (2014) assessed the effect of different sodium hypochlorite concentrations (0.5, 1.5, 3, and 6%) followed by either 17% EDTA or normal saline and reported negative effects of high concentration of sodium hypochlorite on the survival and differentiation of stem cells of the apical papilla (SCAP). They recommended the use of 1.5% sodium hypochlorite followed by 17% EDTA. The use of EDTA following irrigation with sodium hypochlorite is now widely recommended (Wigler et al. 2013). Trevino et al. (2011) assessed the effect of different combinations of irrigants on SCAP and reported the best outcome, in terms of cell survival, was following irrigation with only 17% EDTA.

Therefore the use of 1.5% sodium hypochlorite followed by 17% EDTA is currently the recommended irrigation protocol in RET and should be considered in future studies.

The use of a tri-antibiotic paste containing 100 mg metronidazole, 100 mg minocycline and 100 mg ciprofloxacin has been shown to have sufficient bactericidal efficacy potent enough to eradicate bacteria from the infected dentine of root canals (Hoshino et al. 1996). Recently, minocycline has been eliminated from the mixture due to its potential to discolour the tooth (Kim et al. 2010) which was further supported by unpublished recent work showing a similar antimicrobial effect of the tri-antibiotic and bi-antibiotic pastes.

Achieving a hermetic coronal seal is also crucial in maintaining a sterile root canal environment. The use of MTA in achieving a hermetic coronal seal, hence preventing future contamination, had been associated with crown discolouration. Most commercially available MTA products contain agents used to enhance its radio-opacity, such as bismuth oxide, an agent known to cause discolouration of teeth. Portland cement, which has been shown to cause less tooth discolouration than MTA (Lenherr et al. 2012), is currently used at the Leeds Dental Institute while other materials such as bioceramics or tricalcium silicate cements (Biodentine^®^, Septodont, Lancasted, PA, USA) have been recommended by the American Academy of Endodontic’s clinical considerations for a regenerative procedure.

### Stem cells

There are several sources of stem cells in the oral cavity (Hargreaves et al. 2013) with some researchers implying that stem cells of the apical papilla (SCAP) have a major role in regeneration techniques (Huang et al. 2008). Stem cells populating the root canal system are achieved mainly through induction of bleeding from the periapical area as shown by most techniques published till May 2014 (Kontakiotis et al. 2015). There is some support for this idea especially when a 400–600 fold increase in mesenchymal stem cell markers, in blood collected from the canal, was reported in comparison to levels found in systemic blood samples (Lovelace et al. 2011).

### Scaffolds

Several synthetic and natural scaffolds have been reported in the literature (Murray et al. 2007). Such scaffolds provide three-dimensional structures for stem cells to proliferate and differentiate into the desired odontoblasts. Most RET protocols rely on inducing bleeding into the root canal and subsequent clot formation as a scaffold. The use of a blood clot scaffold, although in principle fulfils the required characteristics of indigenous scaffolds and shown to be successful in promoting continuation of root development, is considered an uncontrolled environment with the potential to promote healing rather than regeneration of desired tissues. Therefore future research for a better scaffold system is crucial for more reliable and consistent results of RET.

### Signalling molecules

Signalling molecules capable of promoting stem cell differentiation are crucial for successful regeneration of pulp tissues. Utilising dentinal wall signalling molecules through the use of 17% EDTA intra-canal irrigation is currently recommended (Trevino et al. 2011). However, there is little evidence that this method results in generating the relevant signalling molecules, which would be useful in triggering the regeneration of tissues required for a successful outcome. If the formation of bone is encouraged, as is likely in the current protocols, the root canal will be filled with bone-like tissue with unintended consequences in the future. Further development of specific signalling molecules capable of promoting odontoblastic cell differentiation, angiogenesis and neurogenesis are required.

### Blood clot formation

Although it is reasonable to expect that a blood clot contains stem cells (Lovelace et al. 2011), scaffolding properties, and signaling molecules, the research underpinning this is very scarce. Furthermore, these factors are not part of a controlled environment and therefore might result in regeneration of unwanted tissues such as bone. Tissue engineering involves the use of controlled processes whereby a suitable scaffold, usually a form of extracellular matrix, is seeded with stem cells and signalling molecules that are known to promote the formation of the desired tissues. Current RETs do not meet this threshold, instead implying unknown and uncontrolled methods of tissue engineering as would be recognised in science. Indeed, histological studies have shown the development of cementum, bone and periodontal ligament cells rather than odontoblasts. This indicates a process of repair rather than a regenerative process usually seen by formation of blood clots (Wang et al. 2010). Such ingrowth of PDL and osteoblasts has been linked to development of internal ankylosis in luxated or avulsed teeth over several years (Andreasen and Andreasen 1994) and therefore long term monitoring of these teeth is needed. The authors advise that if organised pulp-like tissue is desired then in the future any RET should involve the use of more specific scaffolds and signalling molecules that would promote the development of pulp specific tissues including angiogenesis and neurogenesis.

## Damage to Hertwig’s epithelial root sheath (HERS) cells

It is now accepted that the tissues surrounding the apex of an immature permanent incisor tooth in children are rich in stem cells (Lovelace et al. 2011). Significant accumulation of undifferentiated mesenchymal stem cells into the root canal system, when bleeding from the periapical area, has been demonstrated. These stem cells are thought to be mainly stem cells of the apical papilla (SCAP), inflamed periapical progenitor cells (iPAPCs), periodontal ligament stem cells (PDLSCs), bone marrow stem cells (BMSCs) and dental pulp stem cells (DPSCs) (Hargreaves et al. 2013). SCAP and DPSCs were shown to be potent in osteo/dentinogenic differentiation as BMSCs (Huang et al. 2008; Sonoyama et al. 2008).

Although most researchers have focussed on the presence of stem cells, the role of HERS is less well discussed. HERS cells are essential for control of stem cell differentiation in addition to cementum formation as shown by Sonoyama et al. (2007). These authors showed, in vitro, that HERS can promote PDLSCs differentiation into cementum forming cells (Sonoyama et al. 2007). In addition, these authors showed the ability of HERS cells to produce cementum through a process of epithelial-mesenchymal transition, which is described as “a fundamental process whereby cells undergo a developmental switch from a polarised epithelial phenotype to a highly motile mesenchymal phenotype” (Sonoyama et al. 2007). It has been known for a long time that a viable epithelium is essential for the differentiation of the mesenchyme because of its organising influence under which the cells of the mesenchyme differentiate into specialised cells. Therefore damage to HERS cells might prevent differentiation of stem cells into the desired cementoblasts/odontoblasts and therefore preventing continued root formation (Nagata et al. 2014; Saoud et al. 2014).

**Fig. 3:**
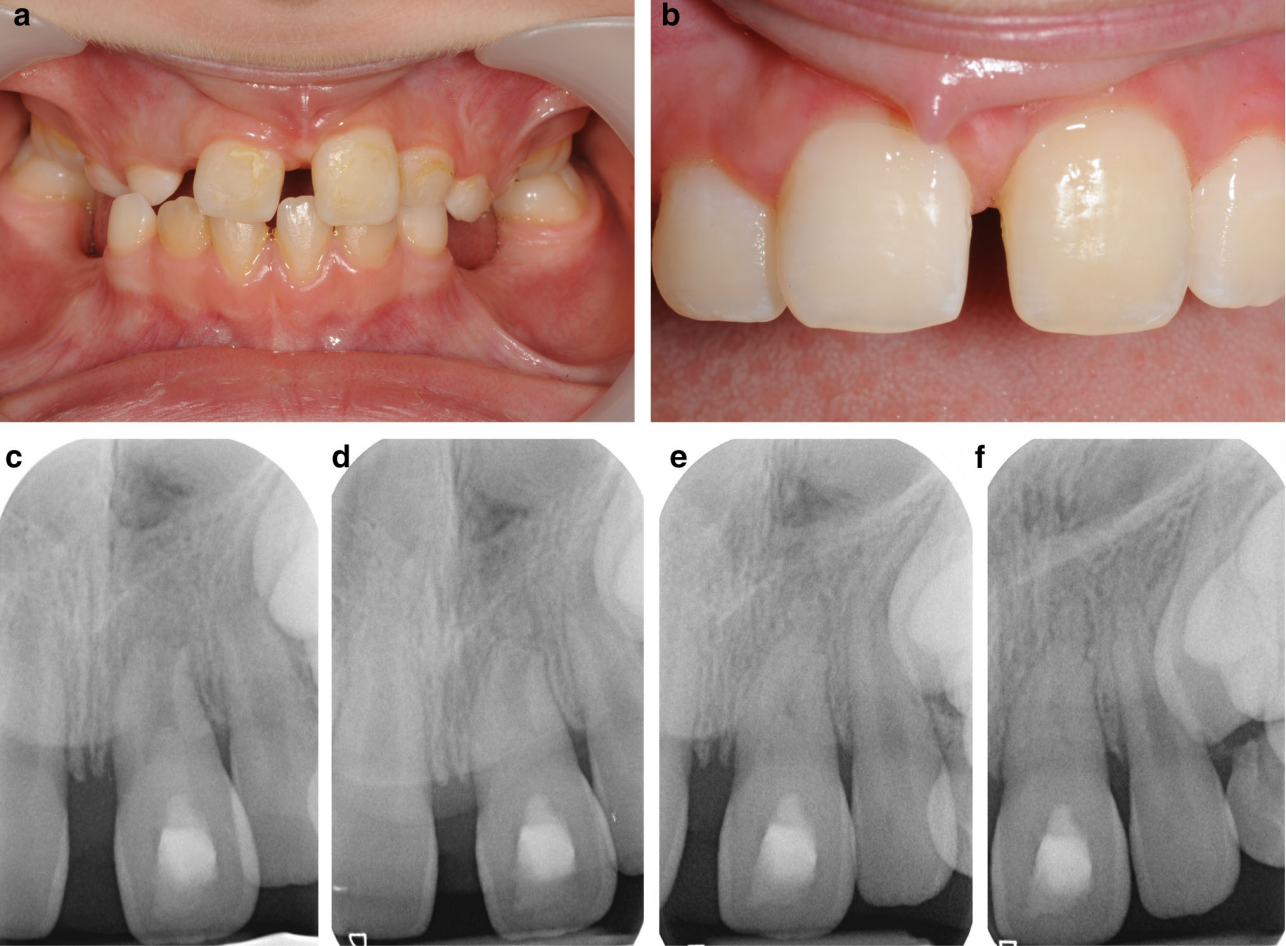
Clinical photographs (**a** before treatment, **b** 2 years following RET) and radiographs (**c** baseline immediately after treatment, **d** 3 months, **e** 9 months, **f** 2 years following RET) of an avulsed and replanted immature 21 treated with RET. No signs of infection were evident after 2 years with continuation of root developmemt, minimal replacement resorption and no signs of infra-occlusion seen at 2 years follow-up

More recently, researchers have tried to differentiate between the causes of loss of vitality on the success of this technique. The success of this technique in terms of continuation of root development and thickening of dentinal walls for teeth that have become non-vital as a result of trauma has been reported (Nagata et al. 2014; Saoud et al. 2014). Saoud et al. (2014) reported higher rates in continuation of root development (41.7%) than those reported by Nagata et al. (2014) (0–5%), however, both showed results at the lower end of those reported for this technique. These results support such a hypothesis that the degree of damage to HERS is an important element in the successful use of this technique (Fig. 3). The case presented in Fig. 3 illustrated an avulsed and replanted immature central incisor (Fig. 3a–f) showing continuation of root development with evidence of minimal replacement resorption. Despite the severe form of trauma, HERS was not severely damaged therefore resulting in continuation of root development. In contrast, the HERS damage was more severe in the case presented in Fig. 2g–l although the tooth sustained only an enamel-dentine fracture. The unpredictable degree of damage to the HERS is therefore likely to affect the results of RET in terms of qualitative increase in root dimensions versus healing.

Furthermore, the induction of bleeding through traumatisation of the apex with a sharp instrument extruded through the apical foramen could prove to be problematic in the long term. The trauma caused by this treatment step could cause further damage to the epithelial cells that form part of the root sheath, so vital for further elongation of the root.

## Radiographic standardisation and reporting methods

One of the main issues discussed by clinicians is the difficulty in standardising radiographic exposures during follow-up appointments. Some researchers used image standardisation softwares such as TurboReg (Bose et al. 2009) in order to align radiographic exposures. These types of software are not only technique sensitive, but also rely on manual identification of landmarks, which is subject to operator error. These softwares, however, might be improved in the near future making their use more meaningful. Others have used bite registration over X-ray positioning devices (Dabbagh et al. 2012). The use of these individualised indices are much easier and more reliable than utilising image alignment software used with different exposures.

Another concern is that different techniques have been reported by various researchers in terms of assessing root development following RET. Some researchers have reported results based on visual assessment by specialists (Ding et al. 2009) while others used digital measurements (Saoud et al. 2014) of root lengths. Current unpublished work, carried out at the Leeds Dental Institute showed a higher inter-examiner reliability with digital measurement techniques [intra-class correlation = 0.974 (95% CI 0.955–0.986)] rather than visual assessment (κ = 0.278) when measuring continuation of root development and thickening of dentinal walls.

### Recommendations

The current techniques used for RET do not fulfil the “gold standard” criteria for engineering a pulp-dentine complex (Murray et al. 2007). The use of a blood clot as a scaffold, although inconsistently successful, may not be the right way forward and further development of RET using scaffolds and appropriate signalling molecules are required. Unfortunately such translational work still requires years of development and apart from treating the tooth with the current RET and assessing the results, there is no way to determine the degree of damage to HERS cells.

It is clear that in cases where loss of vitality has occurred due to dental trauma, all the current regenerative protocols result predictably in good healing of the periodontal tissues but the root development and root thickening remains highly unpredictable. Where loss of tooth vitality is a result of a developmental anomaly and the degree of damage to HERS is possibly minimal, further root development and thickening seems to be seen more often. The profession needs to decide what constitutes a favourable and acceptable core outcome for this treatment approach. If it is felt that healing alone is an acceptable outcome then RET could be more acceptable. However, if healing alone is to be accepted as a good treatment outcome, then this would not solve the problem of root fractures, which are so prevalent in non-vital immature teeth treated with traditional endodontic protocols. So the original premise for which RET was developed is indeed defeated. We feel that all the current evidence and experience in the use of RET points in the direction of the importance of a viable epithelium in the form of HERS. Without the organising influence of the epithelium there would be little chance of the mesenchyme differentiating into the specialist cells that are required for bioengineering the dental pulp. Until such a time that the principles of tissue engineering can be translated into clinical practice, the use of this technique should be limited to only those cases where the prognosis of the immature tooth is deemed to be poor with the use of a traditional approach, such as the use of MTA. In cases where it is indeed decided that RET is the most appropriate treatment approach the following recommendations are made:

1. Further investment in developing scaffolds and signaling molecules.
2. Conducting multi-centre randomised controlled trials comparing RET in managing traumatised versus non-traumatised immature non-vital teeth.
3. There are different RET protocols available in the literature using different sterilisation and coronal seals (Kontakiotis et al. 2015). The Leeds School of Dentistry's RET protocol (Table 2) is based on the best evidence available (to date) and conforms to the American Association of Endodontics’ clinical considerations for regenerative procedures. The main principles of this protocol include:
  - A detailed discussion with the parents and patients should take place explaining the advantages and disadvantages of performing RET as opposed to the MTA apical plug technique.
  - This technique should be avoided in patients where allergy to any of the components, especially to the antibiotics used, exists.
  - The use of individualised bite registration x-ray positioning techniques is recommended to help standardise radiographic exposures in addition to using digital radiography.
  - The irrigation technique should rely on using low concentrations of sodium hypochlorite (NaOCl) as high concentrations have been shown to adversely affect stem cell survival (Martin et al. 2014).
  - The use of 2% chlorhexidine should be discouraged (Trevino et al. 2011).
  - The use of two antibiotics (100 mg metronidazole and 100 mg ciprofloxacin) is as effective as the tri-antibiotic paste and should be considered. Minocycline had been shown to cause tooth discolouration and therefore is not recommended (Sanchez et al. 2004).
  - The coronal seal of an endodontically treated non-vital tooth should be performed using a 3–4 mm layer of Portland cement into the cervical root area of the tooth followed by a glass ionomer layer and composite resin restoration. Portland cement is recommended as an alternative to MTA due to the crown discolouration potential of MTA (Lenherr et al. 2012).
  - 17% EDTA has been shown to be have a positive effect on regenerative procedures and should be used in RET (Trevino et al. 2011).

**Table 2:** The RET protocol used at the Leeds School of Dentistry

First treatment visit
Local analgesia to be given if indicated
The tooth is first isolated using dry dam
The tooth is then accessed and the pulp extirpated using barbed broaches
The canal is then negotiated with minimal or no filing to prevent further weakening of the existing dentinal walls
The root canal system is then irrigated with:
Copious amounts of 1.5% sodium hypochlorite (NaOCl) with the needle introduced into the root canal to a point 2 mm short of the apical foramen in addition to slowly expressing the NaOCl into the periapical tissues
5 mL sterile saline
The canal is then dried using paper points
Metronidazole (100 mg) and Ciprofloxacin (100 mg) should be mixed with distilled water
The mixture of the two antibiotics is then injected into the root canal system
A cotton pellet is then placed to cover the root canal orifice and the access sealed with a glass ionomer cement to prevent any coronal leakage or contamination of the root canal with oral microorganisms
Second treatment visit: (After resolution of infection. If clinical signs or symptoms persist, the procedures performed in the first appointment should be repeated)
Plain local analgesia (no vasoconstrictor) is administered and the tooth isolated and re-accessed as described above
The antibiotic mixture is then flushed out of the root canal by irrigation with copious amounts of normal saline
The root canal is then irrigated with 10 mL 17% EDTA
Following this the root canal should be thoroughly dried with paper points
This is then followed by insertion of a sterile sharp instrument (needle or a finger spreader) to a length of 2 mm beyond the working length, past the confines of the root canal, into the periapical tissues to intentionally induce bleeding into the root canal. The bleeding is then allowed to fill the root canal
Once the root canal is filled with blood, a cotton pledget is placed in the pulp chamber and a clot allowed to form in the root canal
Once the clot has formed, the pulp chamber in the coronal part is thoroughly cleaned to remove any remnants of blood, which could cause discolouration in the future
The access cavity is then hermetically sealed with three layers of material to prevent coronal leakage and contamination: Portland cement, followed by glass ionomer and then composite resin
